# Evaluating nighttime lights and population distribution as proxies for mappinganthropogenic CO_2_ emission in Vietnam, Cambodia and Laos

**DOI:** 10.1088/2515-7620/ab3d91

**Published:** 2019-09-11

**Authors:** Andrea E Gaughan, Tomohiro Oda, Alessandro Sorichetta, Forrest R Stevens, Maksym Bondarenko, Rostyslav Bun, Laura Krauser, Greg Yetman, Son V Nghiem

**Affiliations:** 1 University of Louisville, Department of Geography and Geosciences, Louisville, KY, United States of America; 2 WorldPop, School of Geography and Environmental Science, University of Southampton, United Kingdom; 3 CIESIN, Columbia University, New York, NY, United States of America; 4 Universities Space Research Association, Columbia, MD, USA/NASA, Goddard Space Flight Center, Greenbelt, MD, United States of America; 5 Lviv Polytechnic National University, Lviv, Ukraine; 6 WSB University, Dabrowa Gornicza, Poland; 7 Jet Propulsion Laboratory, California Institute of Technology, Pasadena, CA, United States of America

**Keywords:** greenhouse Gas, CO_2_ emission, uncertainty analysis, emission inventory, nighttime lights, gridded population, Southeast Asia

## Abstract

Tracking spatiotemporal changes in GHG emissions is key to successful implementation of the United Nations Framework Convention on Climate Change (UNFCCC). And while emission inventories often provide a robust tool to track emission trends at the country level, subnational emission estimates are often not reported or reports vary in robustness as the estimates are often dependent on the spatial modeling approach and ancillary data used to disaggregate the emission inventories. Assessing the errors and uncertainties of the subnational emission estimates is fundamentally challenging due to the lack of physical measurements at the subnational level. To begin addressing the current performance of modeled gridded CO_2_ emissions, this study compares two common proxies used to disaggregate CO_2_ emission estimates. We use a known gridded CO_2_ model based on satellite-observed nighttime light (NTL) data (Open Source Data Inventory for Anthropogenic CO_2_, ODIAC) and a gridded population dataset driven by a set of ancillary geospatial data. We examine the association at multiple spatial scales of these two datasets for three countries in Southeast Asia: Vietnam, Cambodia and Laos and characterize the spatiotemporal similarities and differences for 2000, 2005, and 2010. We specifically highlight areas of potential uncertainty in the ODIAC model, which relies on the single use of NTL data for disaggregation of the non-point emissions estimates. Results show, over time, how a NTL-based emissions disaggregation tends to concentrate CO_2_ estimates in different ways than population-based estimates at the subnational level. We discuss important considerations in the disconnect between the two modeled datasets and argue that the spatial differences between data products can be useful to identify areas affected by the errors and uncertainties associated with the NTL-based downscaling in a region with uneven urbanization rates.

## 1 Introduction

Keeping track of spatiotemporal changes of the greenhouse gases (GHG) emissions is key to the successful implementation of the United Nations Framework Convention on Climate Change (UNFCCC) as monitoring emissions directly informs international climate change policy initiatives (Raupach *et al* 2007, Figueres *et al* 2018). While the Paris Climate Agreement recognized the importance of the climate mitigation actions at subnational levels (e.g. cities, private sectors), the emission inventories reported by countries, in the current inventory framework, do not fully allow us to monitor those efforts. This is because the current emission inventory framework is designed to quantify GHG emissions at the national scale with no current, globally consistent requirement for state or city-level emission inventory reporting.

Spatially-explicit emission estimates have been mainly developed for research application purposes, such as atmospheric modeling (e.g. (Andres *et al* 1996, Kurokawa *et al* 2013, Oda *et al* 2018, Janssens-Maenhout *et al* 2019). The spatial extent of emissions is often estimated and/or determined via spatial disaggregation of the emissions estimates made at larger, aggregated scales (e.g. countries and regions) and this is done using geospatial information. The first global CO_2_ map was developed by Anders *et al* (1996) at the Carbon Dioxide Information Analysis Center, (CDIAC) at the Oak Ridge National Laboratory (ORNL). Andres *et al* (1996) used a global population density map from 1984 (Fung *et al* 1991) to disaggregate national level emission estimates to a 1° × 1° grid cell. The approach comes with an inherent assumption of a good correlation between population and fossil fuel carbon dioxide (FFCO_2_) emissions at a large aggregated spatial scale (e.g. state level). More recently, Anders *et al* (2016) updated their approach to include two additional gridded population products to cover more recent time periods (Andres *et al* 2016). Those two products are the Gridded Population of the World v3 (Center for International Earth Science Information Network (CIESIN) and (CIAT) 2005) and LandScan (Dobson *et al* 2000).

Additionally, in response to the strong need for a high-resolution CO_2_ emission map for high-resolution atmospheric modeling and satellite CO_2_ analyses, Oda and Maksyutov (2011) proposed an improved 1 km × 1 km gridded emission model. Known as the Open Source Data Inventory for Anthropogenic CO_2_ (ODIAC), their approach is based on a disaggregation of country level fuel based estimates, such as CDIAC estimates, using the combination of point source information and satellite-observed nighttime lights (NTL). NTL has been identified as a good indicator of human settlement and the intensity of human activities (e.g. (Elvidge *et al* 1999). Other studies have noted limitations for using NTL data as a 1:1 proxy for human population distribution, especially in low-lit, less developed regions of the world (Sutton *et al* 2001, Huang *et al* 2014, Pandey *et al* 2017). However, Oda and Maksyutov (2011) separately map point source emissions that are difficult to approximate by the spatial distribution of NTL and found that NTL is a useful remote sensing tool for disaggregating the remaining CO_2_ emission estimates in timely, updatable manner at a global scale. Because of the high-resolution, updated emissions, ODIAC has been extensively used in atmospheric carbon budget studies across different scales from global (Takagi *et al* 2011, Maksyutov *et al* 2013, Feng *et al* 2017, Crowell *et al* 2019) to urban scales (Ganshin *et al* 2012, Oda *et al* 2012, Brioude *et al* 2013, Lauvaux *et al* 2016, Oda *et al* 2017, Wu *et al* 2018, Reuter *et al* 2019)

While emissions disaggregation using a wide variety of geospatial information is becoming more accessible, errors and uncertainties associated with the resulting emission fields remain fully unquantified. The evaluation of actual errors and uncertainties associated with gridded emissions is challenging fundamentally due to the lack of physical measurements (Andres *et al* 2016, Oda *et al* 2018, 2019). The research community has been studying the use of atmospheric measurements to objectively evaluate emission estimates from reported inventories (top-down analysis, e.g. (Vogel *et al* 2007, Lauvaux *et al* 2016, Nassar *et al* 2017)). However, the ability of constraining emissions highly depends on the atmospheric observation available and is limited to a small area (e.g. city) and particular sources (e.g. power plant). Thus, a common approach has been comparing inventories and using the differences as a proxy for errors and uncertainties in gridded emissions (e.g. (Gately and Hutyra 2017, Hutchins *et al* 2017, Oda *et al* 2018, 2019). Though such comparison does not provide any objective measure regarding the performance of the data used for emission downscaling, it allows for characterizing the differences that are unique to respective inventories (Oda *et al* 2019).

In this study, we evaluate the use of remotely-sensed, nighttime lights as an emission proxy by comparing non-point source ODIAC emissions to population data which are the direct proxy for the intensity of human activities (thus, CO_2_ emissions). We are most interested in comparing gridded CO_2_ emissions and spatially identifying the error and uncertainty of CO_2_ emissions disaggregation techniques as a first step towards improving underlying data sets that inform not only the atmospheric modeling community but also CO_2_ emission estimates at subnational levels. While NTL serves as an excellent proxy for developed countries (e.g. (Oda and Maksyutov 2011)), NTL is thought to perform poorly as a proxy for CO_2_ emission disaggregation in low and middle income countries where the spatial pattern of emission distribution, as associated with population, is not directly proportional to NTL data. Raupach *et al* (2010) demonstrated that by showing correlations between NTL and population, with population acting as a proxy for CO_2_ emissions (Raupach *et al* 2010). Thus, we use the NTL-disaggregated, non-point source CO_2_ emissions from the ODIAC model to compare with a gridded population dataset driven by a set of ancillary geospatial data for 2000, 2005, and 2010. The objective is to examine how spatiotemporal changes in gridded CO_2_ emission estimates based on residential population distributions compare with the changes in gridded CO_2_ emission estimates informed only by NTL data in order to identify areas of uncertainty in the ODIAC model. We examine the difference and similarities of these two datasets at multiple spatial resolutions for three emerging countries in Southeast Asia: Vietnam, Cambodia and Laos. This region represents an area where the NTL performance is poor and emission estimates will increase over time (Schneider *et al* 2015, Fulton *et al* 2017, Pandey *et al* 2017).The three countries also provide an ideal landscape of varying development patterns associated with different brightness intensities and population densities for highlighting areas of uncertainty in the ODIAC dataset.

Population data (POP) are traditionally based on censuses that are linked to areal administrative units of varying sizes, thus the subnational distributions are more constrained than CO_2_ data that is typically based on a country level emission disaggregation (i.e. there are no constraints at subnational level). Some studies have explored the ways to combine the use of NTL and POP data to compensate for the weakness of relying only on NTL for emission disaggregation (Ghosh *et al* 2010, Rayner *et al* 2010). However, the literature lacks an evaluation of the combined use of these two highly-correlated datasets to characterize potential errors and uncertainties associated with the use of the proxy data, such as emission representation errors. We expect that the differences found can be largely explained by the impact of the additional covariates and modeling in the population data, which we consider as potential additions for better CO_2_ emissions mapping.

## 2 Data and methods

### 2.1 CO_2_ emission dataset

The ODIAC is a global high resolution (1 × 1 km) fossil fuel CO_2_ emission data product (Oda and Maksyutov 2011, Oda *et al* 2018). The ODIAC is based on spatial disaggregation of CO_2_ emission estimates made by the Carbon Dioxide Information Analysis Center (CDIAC) at the Oak Ridge National Lab (ORNL) (Boden *et al* 2016). CDIAC emissions are estimated by fuel type (solid, gas, and liquid fuels, bunker fuel, and gas flares) plus cement production, rather than the emission sector that is often used for the national inventory compilation (Marland and Rotty 1984). The ODIAC spatial disaggregation is done in two steps. First, emissions from point sources (mainly power plants) are estimated and mapped using the power plant emission estimates and geolocation taken from a global power plant database. The rest of the emissions (country total minus point source emissions), which we refer to a non-point source emissions, are distributed using the spatial distribution of satellite-observed nightlights (NTL) intensities (Oda and Maksyutov 2011, Oda *et al* 2018). Non-point source emissions are disaggregated to a 1 km × 1 km spatial resolution using Defense Meteorological Satellite Program (DMSP) calibrated radiance NTL data, with mitigated saturation effect, developed by NOAA’s Earth Observation Group (Oda *et al* 2010). The calibrated radiance NTL data are a merged product of the regular DMSP NTL product and benefits from reduced gain observations (Ziskin *et al* 2012). Oda *et al* (2010) show an improved spatial emissions distribution from the original publication by Oda and Maksyutov (2011) due to the use of the calibrated radiance data.

Globally, this emission disaggregation is done for 65 individual countries and 5 aggregated geographical region groups (Oda and Maksyutov 2011). Vietnam, Cambodia and Laos are a part of the Asia Pacific geographical region group in the ODIAC country and region categorization. Since the country emissions for those three countries are once aggregated to the regional total before the emissions disaggregation, the country total emissions do not exactly match with the original CDIAC estimates. However, the geographic aggregation for Vietnam, Cambodia and Laos is necessary for an emission modeling framework that aims at quick, timely updates using the latest fuel statistical data from companies such as BP. Further details of the ODIAC approach and methodology are described elsewhere (Oda and Maksyutov 2011, Oda *et al* 2018). In this study, we excluded point source emissions and only use non-point (diffuse, area) source emissions (total minus - point source emissions) and specific to the study region of interest, focusing on the remotely-sensed NTL, nonpoint source ODIAC emissions. The version of ODIAC used in this study (ODIAC 2017) covers 2000 to 2016 but we use data for the years 2000, 2005 and 2010. The data product is available from http://db.cger.nies.go.jp/dataset/ODIAC/ (Oda and Maksyutov n.d.).

### 2.2 Population dataset

The gridded population data for this study is from the WorldPop Project (www.worldpop.org 2018). The underlying method is one of the more advanced techniques for gridding population distributions which relies on a hybrid approach of using a statistical weighting layer for estimating population density constrained by a dasymetric redistribution of the administrative unit population counts (Stevens *et al* 2015). Such an approach compares favorably or outperforms other techniques for producing gridded population maps (Sorichetta *et al* 2015, Stevens *et al* 2015, Reed *et al* 2018) while recognizing results are dependent on the spatial fidelity and accuracy of the ancillary data and census data used in the model.

The statistical component of the WorldPop model involves training a random forest (RF) model (Breiman 2001) to create a population density layer that will be used to disaggregate the administrative unit-based population counts to a regular grid of fixed spatial resolution. The RF-model is a non-parametric, nonl-inear statistical machine-learning approach that combines a set of decision trees into an ‘ensemble’ learner of multiple trees for a stronger output prediction. The ensemble of models is combined with random sampling of both training observations (bagging) for individual trees and covariate selection during tree growth. The individual trees and their prediction estimates can then be validated against withheld observations (out-of-bag errors, OOB), which can then be used across all trees in the RF to assess both variable importance and prediction performance (Breiman 2001, Liaw and Wiener 2002, Strobl *et al* 2009). The OOB error represents a robust and unbiased measurement of the prediction accuracy of the RF model (Breiman 2001) and a reliable proxy of the accuracy of the final gridded population datasets produced using the RF-based approach (Sorichetta *et al* 2015, Stevens *et al* 2015).

Predictions of population density from the RF model at the pixel level are inherently biased because the model is parameterized at the administrative unit level. Therefore, the pixel level estimates of population density are used as weights to anchor a dasymetric redistribution approach (Mennis 2003) of administrative census counts redistributed to a regular grid of fixed spatial resolution (100 m). Full details on the combined machine-learning and dasymetric modeling technique is found in (Gaughan *et al* 2015, Sorichetta *et al* 2015, Stevens *et al* 2015).

The WorldPop model is parameterized for each year of interest using an aggregated set of administrative units (Laos *n* = 10035, Cambodia *n* = 1621, Vietnam *n* = 688) and by maintaining the same consistent boundary definitions over time (Gaughan *et al* 2016). By relying on census data to inform the modeling process, the gridded population outputs effectively represent residential population counts which is in contrast to data such as Landscan which models ambient population counts (Dobson *et al* 2000). In addition, the 100 m gridded population datasets are eventually spatially aggregated (by summing population per grid cell) and coregistered to match the 1 km spatial resolution outputs from the ODIAC model. Population grids were done for 2000, 2005 and 2010. In terms of the RF model parameterization, subnational population counts for 2000, 2005 and 2010 were either extracted, interpolated, and extrapolated using two census dates (i.e., *t*_0_ and *t_1_*) for all three countries (i.e., 2005 and 2010 for Laos, 1998 and 2008 for Cambodia, 1999 and 2009 for Vietnam). This was done by calculating the growth rate of each administrative unit within each country and applying it to the corresponding population counts (Doxsey-Whitfield *et al* 2015). For each administrative unit, the corresponding exponential growth rate (r) was calculated using the following formula:

$$r=\frac{1}{t}\ln\left(\frac{P_{1}}{P_{0}}\right),$$ (1)

where *r* is the growth rate of a given administrative unit between *t*_0_ and *t*_1_, *P*_0_ is its total population at time *t*_0_, *P*_1_ is its total population at time *t*_1_, and *t* represents the number of years between *t*_0_ and *t*_1_.

**Figure 1:**
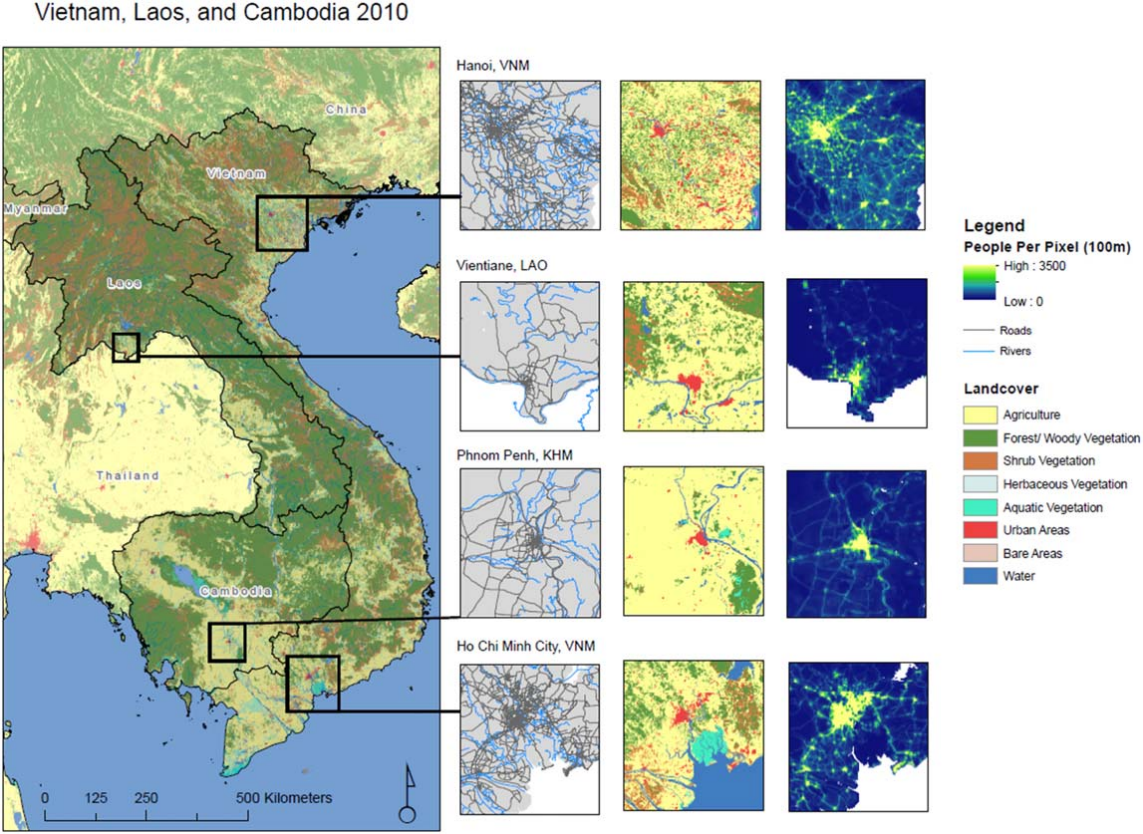
Spatial visualization of rivers, roads and land cover covariates in the nonparametric, ensemble model with sub-panels denoting regions around four large cities in the region. Roads (black) and rives (blue) noted are in the first column panel, ESA land cover in the second column and the 2010 gridded population output in the third column.

Geospatial covariates used as input to the RF have been standardized, harmonized, and co-registered, to the CIESIN Gridded Population of the World v4 archive of administrative-boundaries (http://sedac.ciesin.columbia.edu/downloads/docs/gpw-v4/gpw-v4-country-level-summary-rev10.xlsx) across all three countries of interest. We followed the approach outlined in Gaughan *et al* (2016) and used both temporally-invariant and temporally-explicit covariates (table 1) while excluding NTL data. Temporally-invariant data include topography and slope, average annual precipitation and temperature (representative of the current conditions), presence of main roads and their intersections, waterways, water bodies, and coastlines. Temporally-explicit data, for 2000, 2005 and 2010, include the European Space Agency (ESA) Climate Change Initiative (CCI) land cover layers and presence of protected areas (WDPA 2017). For all temporally-invariant categorical data we produced the corresponding ‘distance-to’ covariate dataset, while for the temporally-explicit categorical data we calculated the distance-to-edge covariates, where distances inside the edge are negative and distances outside the edge are positive. See (Lloyd *et al* n.d.) for the production methodology for how the various covariate datasets were assembled and harmonized. Figure 1 shows the entire study region with land cover, roads, and rivers displayed along with panels of main city area gridded population patterns for 2010.

**Table 1:** Data description for geospatial covariates used in the population model.

Name	Source	Data type and nominal spatial resolution	Data product	Acquisition year
Viewfinder Panoramas	De Ferranti, J, http://viewfinderpanoramas.org/	Continuous raster, 3 arcseconds (~ 100 m at the Equator)	Topography, Slope	~2000
ESA CCI Land Cover Maps – V2.0.7	European Space Adency (ESA) & Université Catholique De Louvain (UCL)	Categorical raster, 10 arcseconds (~300 m at the Equator)	Land cover classes	2000–2015
Open Street Map	OpenStreetMap Foundation (OSMF) & Contributors	Categorical vector	Main roads, main road intersections and waterways	2016
ESA CCI WB v4.0	European Space Agency (ESA)	Categorical binary raster, 30 arcseconds (~ 150 m at the Equator)	Water bodies	2000–2012
WorldClim 2.0	Fick, S.E. and R.J. Hijmans	Continuous rasters, 30 arcseconds (~ 1 km at the Equator)	Mean annual temperature and precipitation	1970–2000
World Database of Protected Areas (WDPA)	UNEP-WCMC	Vector	Terrestrial and marine protected areas	2000–2014
Global Population of the World (GPWv4) Coastlines	CIESIN, Gridded Population of the World v4	Vector	Protected areas	

### 2.3 Relationships between remotely-sensed nightlights and population

To determine the agreement between remotely-sensed NTL and gridded population, we examine the relationship between non-point source CO_2_ emissions and population at multiple administrative levels over the three time points. We then compare the NTL-disaggregated CO_2_ emission estimates (i.e. the ODIAC model) to a disaggregation of the CO_2_ emission estimates by the gridded population (i.e. WorldPop model) for each year. By comparing disaggregations based on NTL versus a simple, per capita disaggregation we can directly interrogate how disassociation between nightlights and population estimates might affect the utility of either approach. Informing the underlying gridded population model is a set of geospatial covariates (table 1) that provide some insight as to the disaggregation of population counts, over space and time, making the population-driven disaggregation approach useful to assess similarities and differences in how NTL, as a singular covariate, redistributes the CO_2_ emission estimates.

To disaggregate by population, we summed the total population count to the national level and created a population proportion layer for each year (e.g. 2010 people per pixel/total population by country). Next, we multiplied the proportion of population through by the total CO_2_ by country for a given year. We did this for the three years of interest (2000, 2005, and 2010) resulting CO_2_ emissions disaggregated by the population model described in section 2.2. We present different metrics to highlight differences in NTL-driven versus population-driven model outputs and identify spatial distributions related to the underlying data informing respective CO_2_ models for each year. In addition, as done by previous studies (Hogue *et al* 2016), we examine the agreement of the two CO_2_ emission maps at different spatial resolutions.

## 3 Results

### 3.1 The NTL model (ODIAC nonpoint source) and the POP model (WorldPop) for 2000, 2005, and 2010

**Figure 2:**
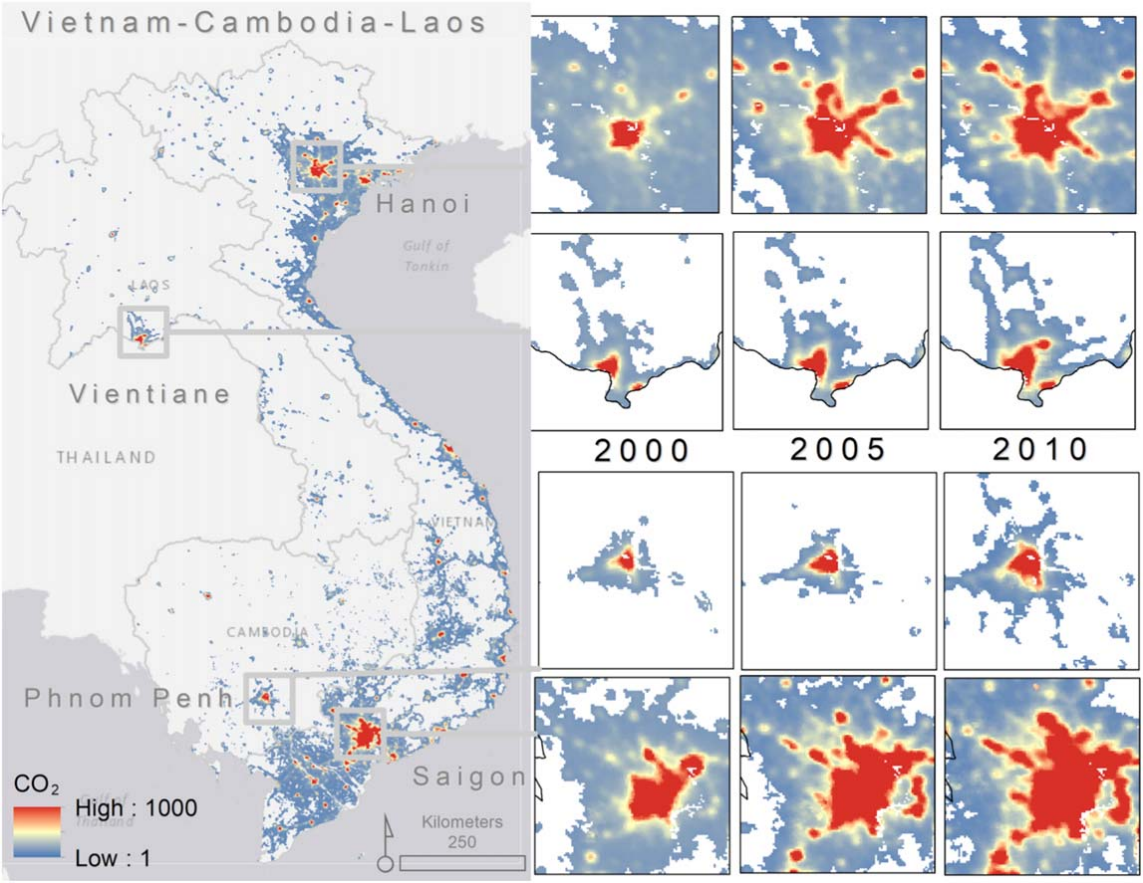
Per pixel estimates of CO_2_ emissions from the ODIAC nonpoint source model. The four largest urbanized regions are highlighted for 2000–2005–2010. The values are given in the unit of metric tonne carbon/year/grid cell (1 × 1 km).

The NTL model indicates expanding source areas for all three countries in a spatially-explicit manner from the 2000–2005–2010 period (figure 2). Vietnam, which is a more urbanized country, shows greater spatial distribution in the estimated CO_2_ emissions as the number of lit grid cells in Vietnam are proportionally higher than in Laos and Cambodia.

**Figure 3:**
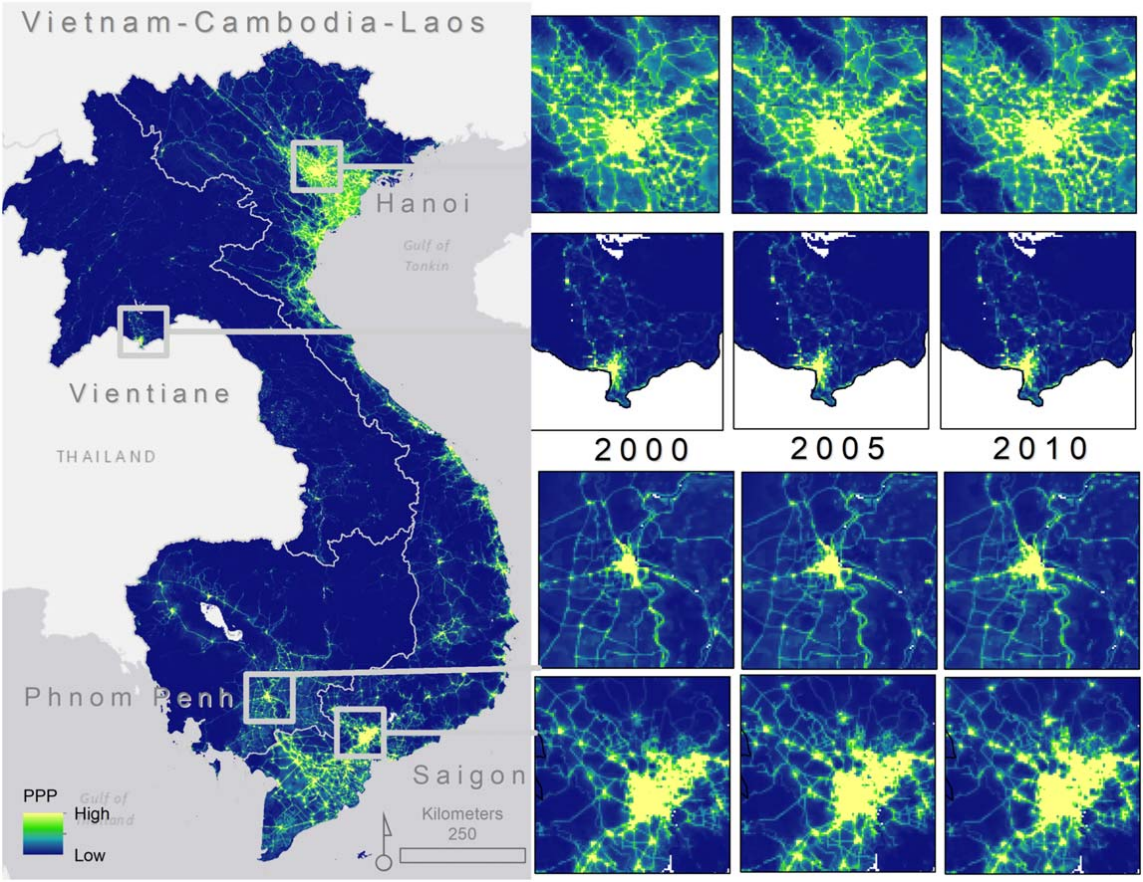
Per pixel counts of people for 2000–2005–2010. The four main urbanized areas are highlighted for each year (1 × 1 km).

Similar to the NTL maps, the annual gridded population data maps show a general increase in population counts over time (figure 3). The percentage of variance explained by the RF model and the prediction error associated to it are equal to 79% and 0.72, respectively, for all three years. The relative importance of a covariate in the RF model is captured by percent increase in mean squared error (%MSE). This means, in the ensemble model prediction phase, that the lower the %MSE, the more important a given covariate will be relative to other covariates when randomly selected and included in a node prediction. Results from the model in this study show that distance to roads and rivers are important to the model in terms of random effects for all three years (figure 1). Agriculture is also important in 2000 and 2005 but becomes slightly less so in 2010 (Supplemental (S1)). Full plot overviews for the model are provided in S1.

### 3.2 Characterizing spatiotemporal change between the NTL-driven model and POP-driven model for CO_2_ emissions

**Figure 4:**
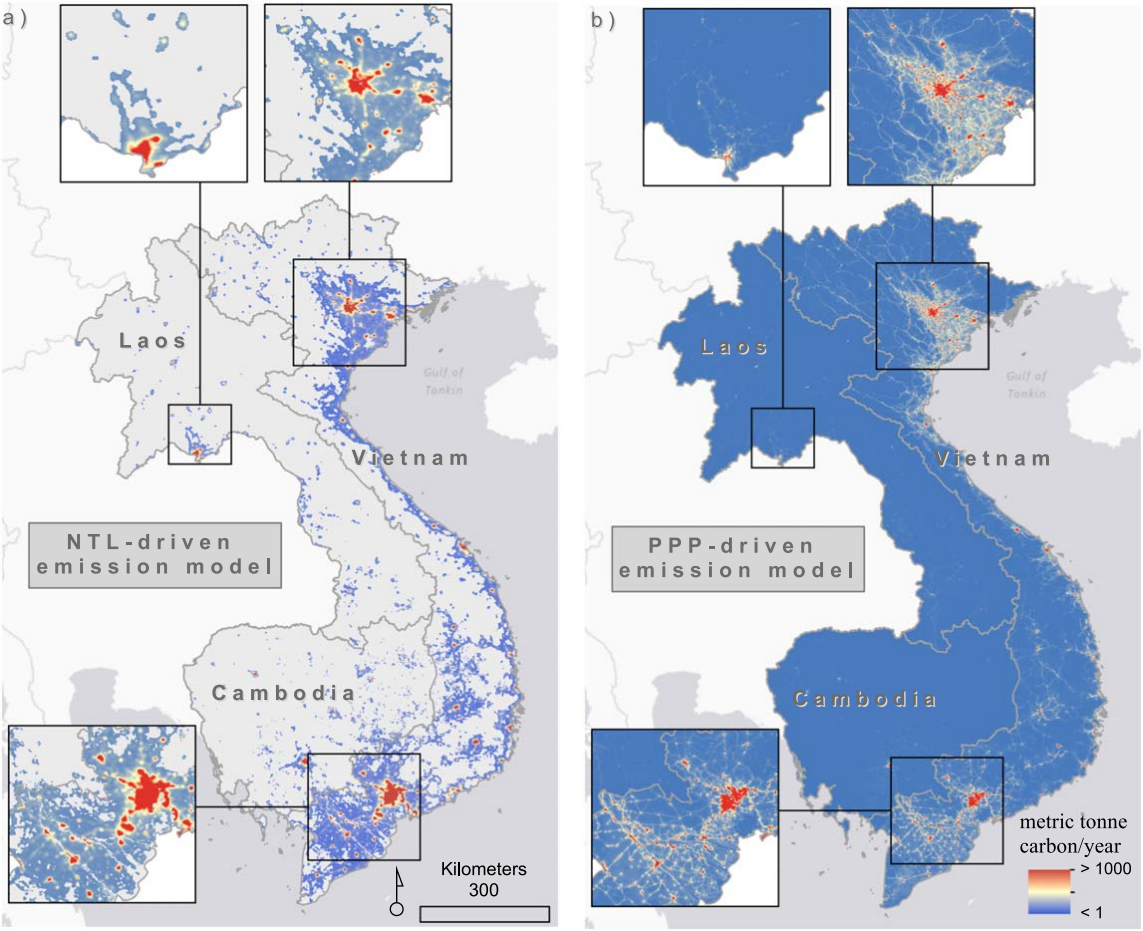
Visual representation of 1 km spatial resolution in 2010 for (a) per pixel estimates of CO_2_ emissions from the NTL-driven model (i.e. ODIAC) and (b) CO_2_ emissions estimates produced using population estimates on a per-pixel basis. The values are given in the unit of metric tonne carbon/year/grid cell (1 × 1 km). In the NTL-driven model (a), the light grey shading within country borders represent grid cell values of 0. There are no grid cell values of zero in the (b) population model.

Figure 4 highlights some important differences in per pixel estimates of CO_2_ emissions from the NTL-driven (i.e. ODIAC) model versus the POP-driven model. The most noticeable difference between figures 4(a) and (b), is that the population-driven model allocates estimates of CO_2_ emission to every grid cell while NTL does not. The gridded population modeling approach may estimate fractional population in a given grid cell (Stevens *et al* 2015) and will not predict zero in grid cells unless the administrative unit contains zero people. The type of approach performs favorably, and in many cases outperforms, other types of gridded population modeling approaches (Reed *et al* 2018), which may translate into better gridded residential CO_2_ products. The NTL-driven model includes zero as a possible grid cell value (i.e. grey-shaded pixels in figure 4) and thus CO_2_ emissions are more concentrated into certain areas for the NTL-driven model rather than spread across the entire study area.

**Figure 5:**
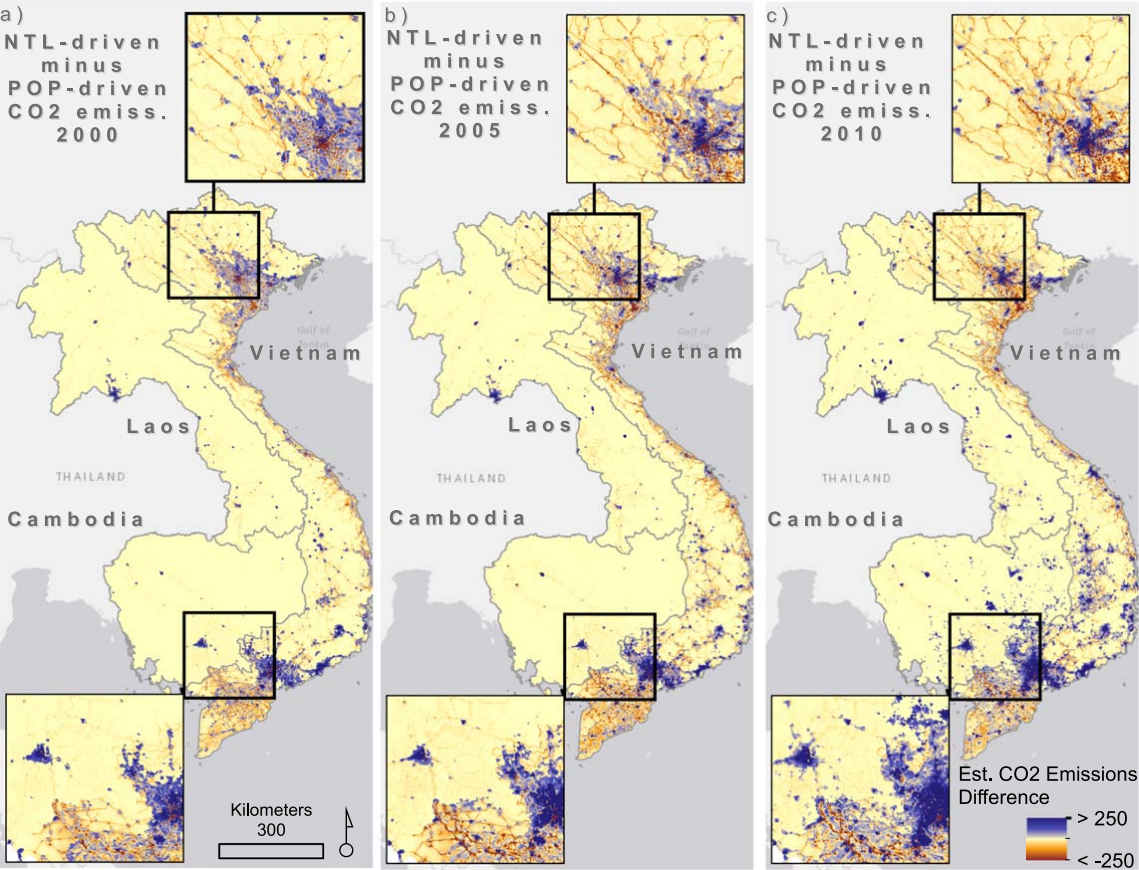
Per pixel differences in CO_2_ emissions estimates produced using only nighttime light intensity, minus those produced using population estimates (per capita emissions). Units are expressed in tonne carbon/year/grid cell and results are separated by the years 2000 (a), 2005 (b), and 2010 (c).

When we difference the NTL-driven model estimates from the POP-driven model for disaggregating CO_2_ emission estimates, the regional biases in the CO_2_ emission from the NTL-driven model are more apparent. In figure 5, positive differences indicate grid cells where the NTL-driven model estimates higher emissions and negative differences indicate areas where population-based CO_2_ emissions estimates are higher than NTL-based estimates. Thus, figure 5 illustrates that through time, nighttime lights-based emissions disaggregation tends to concentrate those estimates in different ways than population-based estimates. It is clear that population-driven model of emissions estimates, which excludes nighttime lights from ancillary data sources, tends to estimate higher emissions outside of the highly urbanized and developed areas of Vietnam, Cambodia, and Laos (shown by the orange-red tones in figure 5). These tend to be areas along major roads, intersections, and where population has been counted in census data but where nighttime lights may not be present. However, across 2000–2010 we see an expansion in the concentration of CO_2_ source area estimates due to an increase in nighttime lighted areas most likely associated with development, as noted by the increase in ‘purple’ grid cells from 2000–2005–2010.

**Figure 6:**
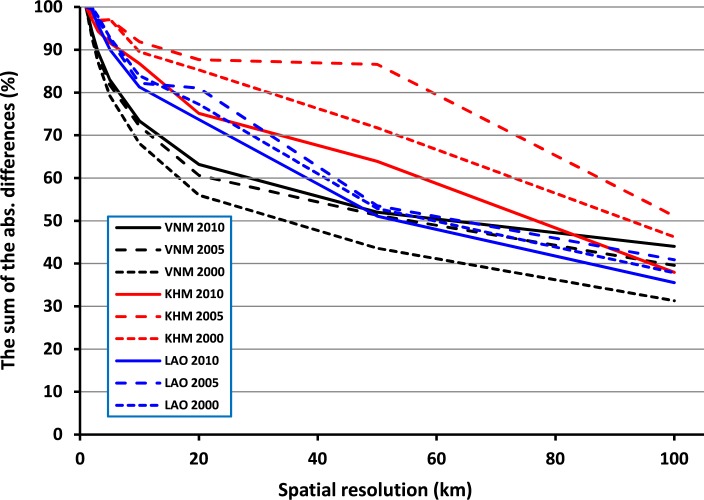
The level of disagreement between the two gridded CO_2_ datasets by the spatial resolution (pixel size) in km versus the sum of the absolute differences at the spatial resolution divided by the sum of the absolute differences at 1 km, presented as a percentage.

Figure 6 shows the level of disagreement between the two gridded CO_2_ datasets by spatially aggregating at multiple spatial resolutions (i.e. by coarsening the two 1 km gridded datasets to 2, 3, 5, 10, 20, 50 and 100 km). As done in Oda *et al* (2019), we calculated the sum of the absolute differences at grid levels and defined the initial difference at 1 km as 100%. The results provide a means to assess how quickly the various datasets for each country and year converge on similar patterns regardless of what data informs the CO_2_ disaggregation. At the country level, while decreasing the spatial resolution of the two datasets especially at the first 25 km or so, there is a sharper gradient in the decrease of disagreement for Vietnam than for Laos and Cambodia. This suggests that, in general, there is a stronger correlation between the NTL and population spatial pattern in Vietnam than in the other two countries (with a better correlation in Laos than Cambodia), and that this correlation is stronger at lower spatial resolutions. In other words, the coarser the spatial resolution of the gridded CO_2_ datasets, the more similar the outputs that are obtained by aggregating the NTL-based and the POP-based CO_2_ grid.

Temporally, by decreasing the spatial resolution of the two gridded CO_2_ datasets for Vietnam, the disagreement is lowest for 2000 than for 2005 and 2010. Quite the opposite is observed for Laos and Cambodia, with the disagreement between the two corresponding gridded CO_2_ datasets generally lower for 2010 than for 2000 and 2005. In other words, while for Vietnam the outputs obtained by aggregating the NTL-based and POP-based CO_2_ grids decrease ‘faster’ overall compared to Cambodia and Laos, the pattern across the different years suggests Vietnam has a different pattern of development and land use, which is reflected by the associations of NTL to population across time. We also note that the differences did not converge to zero. At a coarser resolution, the sum of differences is not largely impacted by the differences in small spatial patterns. We interpret the remaining differences at coarse spatial scale as a proxy bias due to the use of the NTL.

### 3.3 Relationships between the ODIAC-based CO_2_ emissions and WorldPop population distribution for multiple subnational levels

To examine different spatial aggregations of the CO_2_ emission estimates for 2000, 2005, and 2010, we sampled the NTL-based ODIAC and WorldPop population distribution outputs using different administrative unit level boundaries (i.e., *n* = 11,163 (level 3), *n* = 678 (level 2), and *n* = 63 (level 1) administrative units) and show the correlation coefficients for each level, by year, in table 2. The results highlight expected patterns for Vietnam, with improved correlation over time and improved correlation for coarser administrative unit levels (1st level is better than 2nd level, and better than 3rd level). This is consistent with results shown in figure 6 noting less disagreement when data sets are spatially coarsened. Laos and Cambodia show some deviation from this rule which we discuss further below. In addition, we plot the percent shares in the county total population to the NTL-based emissions from the ODIAC model which is located in the S1.

**Table 2:** Correlation coefficients between the spatial aggregations of the CO_2_ emission estimates based on the NTL-based ODIAC and World pop population distribution outputs for 2000, 2005, and 2010.

Administrative level	Number of units	2000	2005	2010
	Vietnam			
1st	63	0.857	0.877	0.881
2nd	678	0.665	0.709	0.729
3rd	11,163	0.585	0.653	0.708
	Laos			
1st	18	0.548	0.567	0.675
2nd	142	0.595	0.566	0.663
	Cambodia			
1st	25	0.502	0.544	0.409
2nd	178	0.548	0.653	0.431
3rd	1,576	0.419	0.471	0.245

We also examined the numbers of the administrative units that NTL failed to allocate CO_2_ emissions for (administrative units with zero emissions) (S1). At the finest administrative level for each country, the numbers of the administrative units consistently decreased especially from 2005 to 2010. For example, 2,847 administrative units in Vietnam in 2000, which accounted for 54% of the total source area (approximated by the sum of admin unit areas) and housed 15% of the total population, were not identified by NTL (zero emissions allocated). In other words, using NTL as the only proxy data for disaggregation failed to identify those 15% of the population and the their CO_2_ emissions were misallocated somewhere else. However, we believe these emissions might not be significant as this 15% people’s activity do not seem to be CO_2_ intensive (no lights, or not developed). In 2010, 31% of the source region remains zero emissions, but that only accounts for 6% of the population. The numbers gradually decreased to 42% in 2005 and then 31% in 2010. Cambodia showed a drastic change from 92% in 2000, 90% in 2005 and then 46% in 2010. Laos also showed the change in the similar way (62% in 2000, 63% in 2005 and 30% in 2010). This analysis is meaningful as this is not impacted by the differences in spatial patterns and population is constrained at these administrative levels. We present this as a loose estimate of error associated with NTL-based emission downscaling at the administrative unit level.

## 4 Discussion

Inherent in gridded emissions data like ODIAC are at least two big sources of uncertainties: (1) total emission errors and (2) spatial disaggregation errors. The analysis in this paper focuses on better understanding sources of spatial errors as it relates to subnational estimates and its direct relevance on climate mitigation policy. To examine the subnational level, we leverage another gridded data product, population data, to examine the uncertainties with the ODIAC model. The population estimates use source data that traditionally stems from censuses or surveys. That tabular information is then linked to irregular and varying sized administrative units for subnational spatial representation (Tatem *et al* 2012, 2014) and provides the source input for gridded population models. There will be inherent error in the gridded population data due to the uncertainty in the input data and the method and scale of model parameterization (Heuvelink 1998). However, there is still value in comparing the different data sources, in this case, the NTL-driven ODIAC model and the WorldPop population data, for estimating gridded CO_2_ emissions and identifying areas of potential uncertainty in the ODIAC model.

At the various administrative unit levels, Vietnam shows the highest correlation between the distribution of lights and population (table 2, figure S5 is available online at stacks.iop.org/ERC/1/091006/mmedia), with the correlations improving across time. A higher correlation between the ODIAC model and population exists from 2000 to 2005 to 2010 due to increased development associated with more ‘lit’ and highly populated grid cells. (Hogue *et al* 2016, Oda *et al* 2019). In Laos and Cambodia we see low correlations across different administrative levels, with inconsistent trends temporally in their degree of association. We hypothesize this may relate to the concentration of both population and CO_2_ emission estimates to the more urbanized areas for those countries and that there was strong population growth patterns outpacing changes in the emission processes during the time period analyzed (2000–2010) but further inquiry is needed to test those ideas.

For Vietnam, the fact that there is a sharper gradient of disagreement between the two gridded CO_2_ datasets, especially in the first 25 km, for 2000 than for 2005 and 2010 (figure 6) could be due to the fact that the actual correlation between NTL and population distribution, at least at the 1 km grid cell level, is decreasing over time. This may be explained by the increasing use of lights for agricultural and aquacultural purposes (Chi Ling *et al* 2009). In other words, over time, lighted grid cells become increasingly associated to unpopulated grid cells corresponding, for example, to dragon fruit plantations and/or aquaculture installations (Kumari *et al* 2016). Conversely, in Laos and Cambodia, the disagreement between the two gridded CO_2_ datasets decreases ‘faster’ for 2010 as the data is spatially coarsened than for the other two points in time suggest that the correlation between NTL and population distribution at the grid cell level is improving over time. One possible explanation might be due to faster rates of development-related electrification and urbanization-related population densification than Vietnam (Organisation for Economic Co-operation and Development 2018, United Nations 2019).

In figure 5, we see minimal differences in the use of the NTL data versus the population data as proxies for estimating subnational CO_2_ emissions in less populated, more rural areas. However, the two proxies spatially and temporally differ in their disaggregation of CO_2_ emissions for other areas due to the fact that the correlation is not always high between NTL and human residence. Interestingly, the NTL-based disaggregation tends to produce higher CO_2_ emission estimates in more densely populated areas compared to the population-based disaggregation technique which is more liable to allocate a lower value in densely populated environments (Deville *et al* 2014, Dijkstra and Poelman 2014).

For the ODIAC model, the non-point source estimates from national CO_2_ emission inventories are disaggregated solely based on NTL brightness, as captured by the DMSP-OLS sensor (Oda and Maksyutov 2011, Oda *et al* 2018). There are no subnational constraints imposed on the process of redistributing CO_2_ emission estimates to 1 km × 1 km grid cells. For the corresponding population-driven estimate of CO_2_ emissions, the underlying population model is parameterized based on subnational administrative units, along with a set of geospatial covariates, which influences the redistribution of CO_2_ emission estimates in for each year.

The gridded population model has a more complex technique for weighting the disaggregation for nonpoint source estimates into 1 km × 1 km grid cells. By using an ensemble of trees, the RF approach provides flexibility for the type of data and type of relationship between predictor and response variables (section 2.3). NTL data is one possible option for inclusion in the set of covariates of the RF model, although it was excluded from the current model due to endogeneity concerns in comparing to the ODIAC data. The second part of the model, the dasymetric constraint, is based on subnational totals for population. Thus, the population model has two levels of additional information for the redistribution of non-point source estimates—(1) a pixel-level population density weighting from the RF model and (2) a subnational constraint from administrative unit totals.

Ultimately, the reliability of the gridded population data is a function of the input population counts, which will vary on a country basis, the reliance of the use of ancillary variables with inherent error, and spatial grain of the subnational constraint applied in the model (Sinha *et al* 2019). There are multiple methods in the literature for modeling gridded population, including those datasets used in other gridded emissions studies (Leyk *et al* 2019). Each technique has its own pros and cons but consistency in the considerations for modeling across space and time is key for reliable comparisons of the gridded population products and any subsequent applications such as CO_2_ emissions disaggregation models.

That said, the use of a gridded population product that has a set of underlying covariates informing the estimates for population distribution provides a useful assessment metric for examining error and uncertainty in the ODIAC model. Nighttime lights (NTL) will always be an imperfect proxy for capturing non-point source emissions associated for FFCO_2_, and as past research has indicated, the knowledge of where people live in addition to NTL has potential to spatially refine these estimates (Oda *et al* 2019). It is particularly important, therefore, that further research leveraging finer spatial resolution emissions data, where available, be used to explore the uncertainty and sensitivity of these and similar approaches to CO_2_ disaggregation.

## 5 Summary

Recognizing the different modeling methods that inform data proxies in the disaggregation of national CO_2_ emissions estimates has important implications for the CO_2_ modeling community. Findings from this study highlight differences in the NTL and population datasets to estimate subnational CO_2_ emissions for a region where development and shifts in population distribution are uneven from 2000–2010 with varying growth trajectories that are country and region specific.

Geospatial data-driven techniques to disaggregate emissions is an important step forward for policy-relevant data products, even while recognizing challenges that exist in reconciling spatial and temporal considerations of data type, model application and methods used in analysis. However, more work is needed to identify the effective spatial resolution for such data and recognizing the underlying assumptions and data constraints of the geospatial proxies is paramount for judging the correct proxies to use in analysis. CO_2_ gridded data are often used as an input for atmospheric CO_2_ modeling. Depending on the problem setting, the spatial resolution of the modeling varies greatly from 1 km to much coarser spatial resolutions. Finally, considering that emission inventories are constructed for different compounds in a systematic way, the considerations of this study are applicable to other compounds such as air pollutants from fuel combustion.
